# Autophagy Induces Expression of IL-6 in Human Periodontal Ligament Fibroblasts Under Mechanical Load and Overload and Effects Osteoclastogenesis *in vitro*

**DOI:** 10.3389/fphys.2021.716441

**Published:** 2021-08-27

**Authors:** Alexandra Mayr, Jana Marciniak, Benedikt Eggers, Kim Blawat, Jan Wildenhof, Rogerio Bastos Craveiro, Michael Wolf, James Deschner, Andreas Jäger, Svenja Beisel-Memmert

**Affiliations:** ^1^Department of Orthodontics, Center of Dento-Maxillo-Facial Medicine, University of Bonn Medical Center, Bonn, Germany; ^2^Department of Oral, Maxillofacial and Plastic Surgery, Center of Dento-Maxillo-Facial Medicine, University of Bonn Medical Center, Bonn, Germany; ^3^Private Clinic Schloss Schellenstein, Olsberg, Germany; ^4^Department of Orthodontics, Faculty of Medicine, University Hospital Aachen, Aachen, Germany; ^5^Department of Periodontology and Operative Dentistry, University Medical Center of the Johannes Gutenberg University, Mainz, Germany

**Keywords:** autophagy, cell-cell communication, IL-6, ADAM10, ADAM17, mechanical load, mechanical overload, human periodontal ligament fibroblasts

## Abstract

**Objective:** Autophagy is an important cellular adaptation mechanism to mechanical stress. In animal experiments, inhibition of autophagy during orthodontic tooth movement triggered increased expression of inflammation-related genes and decreased bone density. The aim of this study was to investigate how autophagy affects cytokine levels of interleukin 6 (IL-6) in human periodontal ligament (hPDL) fibroblasts under mechanical pressure and the resulting influence on osteoblast communication.

**Methods:** hPDL fibroblasts were subjected to physiologic mechanical load, constant overload, or rapamycin treatment for 16 to 24 h ± autophagy inhibitor 3-MA. Autophagosomes were quantified by flow cytometry. Gene expression of *il-6* as well as IL-6 levels in the supernatant were determined with rtPCR and ELISA. To investigate the influence of mechanically-induced autophagy on cell-cell communication, an osteoblast-culture was subjected to supernatant from stimulated hPDL fibroblasts ± soluble IL-6 receptor (sIL-6R). After 24 h, *osteoprotegerin* (*opg*) and *receptor activator of nuclear factor* κ*B ligand* (*rankl*) gene expressions were detected with rtPCR. Gene expression of *a disintegrin and metalloproteinases* (*adam*) *10* and *17* in stimulated hPDL fibroblasts was examined via rtPCR.

**Results:** Autophagy was induced by biomechanical stress in hPDL fibroblasts in a dose-dependent manner. Mechanical load and overload increased IL-6 expression at gene and protein level. Autophagy inhibition further enhanced the effects of mechanical stimulation on IL-6 expression. Mechanical stimulation of hPDL fibroblasts downregulated *adam10* and *adam17* expressions. Inhibition of autophagy had stimulus-intensity depending effects: autophagy inhibition alone or additional application of physiological stress enhanced *adam10* and *adam17* expressions, whereas mechanical overload had adverse effects. Osteoblasts showed significantly reduced *opg* expression in the presence of supernatant derived of hPDL fibroblasts treated with autophagy inhibitor and sIL-6R.

**Conclusion:** IL-6 levels were increased in response to pressure in hPDL fibroblasts, which was further enhanced by autophagy inhibition. This caused a decrease in *opg* expression in osteoblasts. This may serve as an explanatory model for accelerated tooth movement observed under autophagy inhibition, but may also represent a risk factor for uncontrolled bone loss.

## 1. Introduction

Orthodontic tooth movement (OTM) is based on the principle that teeth are moved through bone. To assess processes involved in OTM at molecular level the interplay of two key tissues, periodontal ligament (PDL) and alveolar bone, needs to be examined.

The PDL, a connective tissue in which PDL fibroblasts account for approximately 50–60% of the total cell number, has two main functions: first, the transmission and absorption of mechanical load, and second, providing vascular and nutrient supply to the cementum, the alveolar bone and the PDL itself (Storey, 1973; McCulloch and Bordin, 1991). According to the “pressure-tension theory” the PDL responds to changes in mechanical loading within the bony socket. The theory proposes that PDL fibroblasts play an important role in osteoclastic resorption and/or osteoblastic apposition in accordance with the mechanical stimulus (Sandstedt, 1904; Oppenheim, 1911; Schwarz, 1932). Thus, OTM is induced by application of mechanical forces to the periodontium, resulting in a biomechanical load on cells and tissues, which is subsequently followed by a sterile inflammation involving the release of multiple inflammatory cytokines including interleukin-6 (IL-6) (Li et al., 2018).

IL-6 is a cytokine with important functions in the body's systemic defense response to injury. However, its functions also include influencing bone metabolism. It is produced by various cell types (Garman et al., 1987; Lotz et al., 1988; Gauldie et al., 1992; Ogawa, 1992). For interaction of IL-6 with its target cell, there is a classical signaling pathway and a trans-signaling pathway. The classical pathway is only important in specific cell types, such as macrophages, neutrophils, hepatocytes and some type of T-cells, whereas the trans pathway has a widespread occurrence (Scheller et al., 2011). However, activation of the trans pathway is dependent on the presence of sIL-6R, which is, among others, cleaved from cell surfaces by specific A Disintegrin And Metalloproteinases 10 and 17 (ADAM10 and ADAM17) (Müllberg et al., 1994; Matthews et al., 2003; Chalaris et al., 2007, 2010). However, the tissue reaction is dependent on the amount of biomechanical load – whereas moderate pressure is an integral part of periodontal tissue homeostasis, mechanical overload leads to cell damage associated with an increased PDL fibroblasts death rate and dysregulated bone remodeling (Tang et al., 2006; Marchesan et al., 2011; Blawat et al., 2020).

Remodeling of the alveolar bone is performed by two different types of cells – osteoblasts and osteoclasts. Osteoblasts constitute bone apposition but also control osteoclast differentiation (Karsenty, 2003). Osteoclasts are responsible for degradation of the organic matrix through dissolution of minerals in an acidic environment as well as enzymatic resorption. These multinucleated cells are formed by the union of multiple monocytic precursors (Teitelbaum, 2000; Boyle et al., 2003). Osteoblasts promote osteoclast differentiation by expression of receptor activator of nuclear factor κB ligand (RANKL) on the surface, which interacts with receptor activator of nuclear factor κB (RANK) expressed on osteoclast progenitor cells (Udagawa et al., 1995; Palmqvist et al., 2002). However, they also express osteoprotegerin (OPG), a decoy receptor for nuclear factor κB ligand (RANKL). By binding RANKL, OPG inhibits the activation of NF-κB, a central and fast-acting transcription factor for immune activation and differentiation of precursor cells into osteoclasts (Theoleyre et al., 2004). Cytokines, especially IL-6, have been reported to influence the RANK-RANKL-OPG system in favor of osteoclast differentiation (Udagawa et al., 1995).

Autophagy, the process of self-consumption, has already been proven to be an important process during OTM. In rodents, inhibition of autophagy during mechanically induced tooth movement increased the gene expression of inflammatory markers and decreased alveolar bone density thereby accelerating tooth movement (Chen and Hua, 2021). Autophagy is essential for adaption to cellular stress, including nutrient, hypoxic, inflammatory and mechanical stress signals (King, 2012; Antonioli et al., 2017). In dependence of the stress threshold, autophagy controls cell fate; it can ensure cell survival, but also induce cell death (Mariño et al., 2014). In previous studies we emphasized the importance of autophagy for periodontal stress regulation. Autophagy was induced by tensile strain as well as by pressure in PDL fibroblasts in a dose-dependent manner (Memmert et al., 2019, 2020; Blawat et al., 2020). The modulation of autophagy by cytokines is known and widely studied. Th1 cytokines are suspected to function as inducers of autophagy, whereas Th2 cytokines act as autophagy inhibitors. The downstream influence of autophagy on cytokine expression on the other hand has been much less studied so far. However, how exactly autophagy interferes with periodontal stress regulation and the subsequent effects on cell-cell communication are still unknown, although we know from different cell types that autophagy affects cytokine expression and therefore cell communication (Harris, 2011; Lapaquette et al., 2015).

Therefore, this study aimed to investigate how autophagy affects cytokine expression of PDL fibroblasts under biomechanical load and overload, with a special focus on IL-6 expression. Furthermore, the impact of autophagy on PDL fibroblast communication with osteoblasts was studied with emphasis on osteoclastogenesis.

## 2. Materials and Methods

### 2.1. Culture and Treatment of Cells

Human PDL (hPDL) fibroblasts were purchased in second passage (Lonza, Basel, Switzerland) and cultured in 75 cm^2^ cell culture flasks (Cellstar^Ⓡ^, Greiner Bio-One, Frickenhausen, Germany) in Dulbecco's minimal essential medium, supplemented with 10% fetal bovine serum (FBS), 100 units/ml penicillin and 100 μg/ml streptomycin (all purchased from Invitrogen, Karlsruhe, Germany) at 37°C in a humidified atmosphere of 5% CO_2_. Medium was changed every 3–4 days. For experiments, hPDL fibroblasts were used between passages three and five. 100,000 cells per well were seeded on ultra-thin gas-permeable cell-culture dishes (lumox^Ⓡ^ dish 35, Sarstedt, Nümbrecht, Germany), which were previously coated with attachment factor (Biologics Attachment Factor 1x, Gibco, Thermo Fisher Scientific, Waltham, Massachusetts, MA, USA), and grown to 80% confluence. One day prior to stimulation, FBS concentration was reduced to 1%.

After approval of the Ethics Committee of the University of Bonn and written informed consent by the patients or legal guardians (#117/15 Memmert), primary human alveolar osteoblasts (phAOBs) were explanted from alveolar bone fragments, which would have been discarded otherwise. These alveolar bone fragments were obtained of donors (*n* = 3), who underwent extractions of third molars.

**Figure 1:**
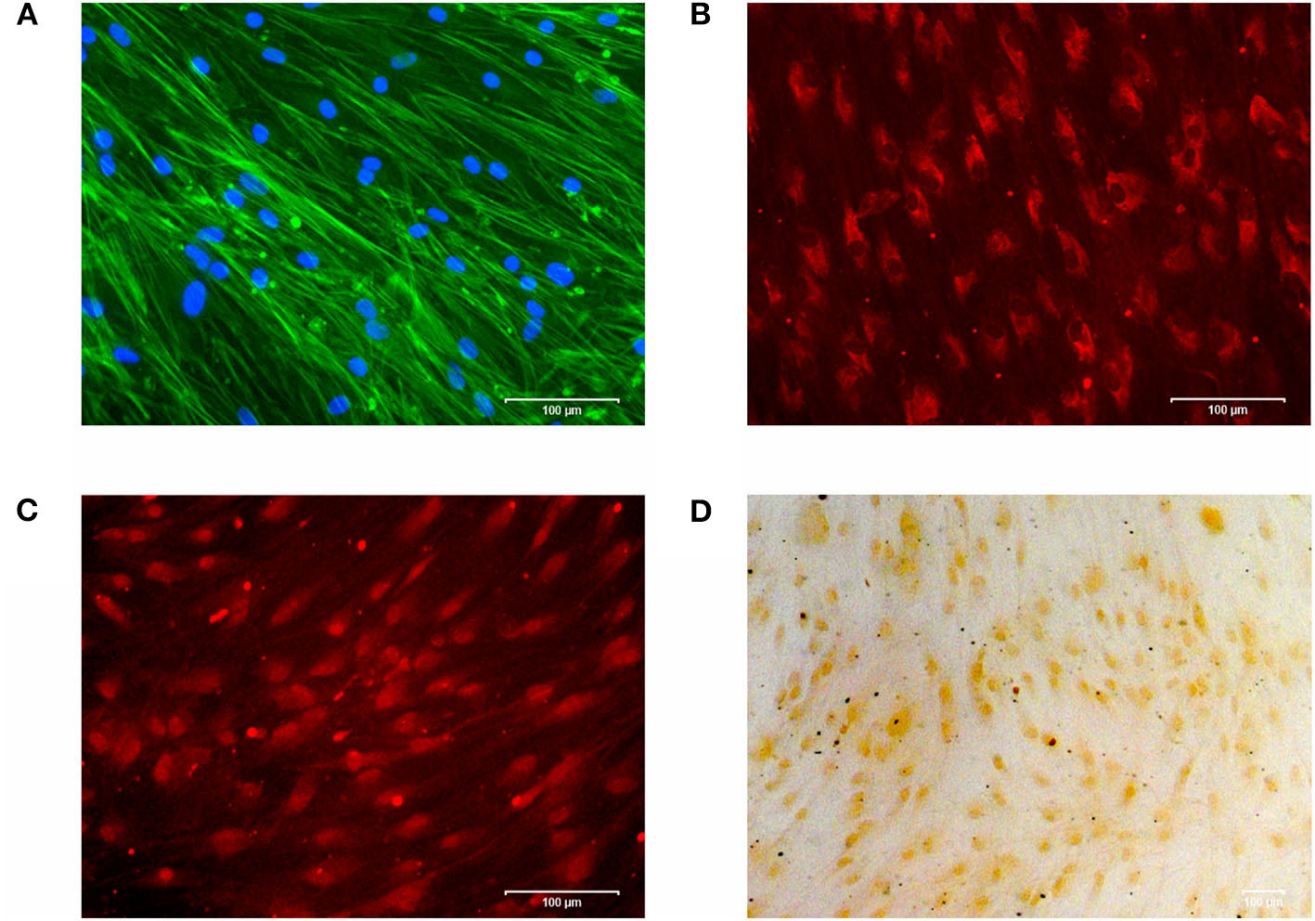
**(A)** Cytoskeleton and cell viability of phAOBs were confirmed by DAPI/Phalloidin staining. **(B)** Expression of type I collagen visualized by immunofluorescence. **(C)** Immunofluorescence staining of osteocalcin. **(D)** Positive mineralization was confirmed by alizarin red staining. Scale bars indicate 100 μm. Experiments were performed in triplicates and representative images of cells from one donor are shown.

For cell characterization, cells were cultured on cover slips in osteogenic medium (DMEM supplemented with 1% FBS, 1% penicillin/streptomycin, 10 nM dexamethasone, 280 μM ascorbic acid and 5 mM β-glycerophosphate (Sigma-Aldrich, Munich, Germany). The medium was replaced every 3 days. After 28 d, cells were incubated with 4% paraformaldehyde (Merck, Darmstadt, Germany) for 20 min and 0.05% Triton^Ⓡ^ X-100 (Merck) for 5 min to fix the cells for subsequent staining. Cell morphology and cell viability was confirmed by a double DAPI/Phalloidin staining: cell monolayers were incubated with fluorescent conjugates of Phalloidin (Sigma-Aldrich, 100 μM) for 40 min, followed by DAPI working solution (Sigma-Aldrich, 1 μg/ml) for 5 min. Stained cells were analyzed with the ZOE^*TM*^ Fluorescent Cell Imager (Bio-Rad, Hertfordshire, Great Britain) (Figure 1A).

For immunocytochemistry, cells were blocked with 5% bovine serum albumin fraction V (Roche Diagnostics, Indianapolis, USA) in 1x PBS for 40 min followed by overnight incubation with primary antibodies: monoclonal anti-collagen I antibody ab21286 (rabbit, Abcam, Cambride, UK) and monoclonal anti-bovine osteocalcin antibody M041 (mouse, TaKaRa Bio Europe, Saint-Germain-en-Laye, France). After rinsing with 1x PBS, secondary antibodies Goat pAb to Rb igG (CY3) ab6939 (Abcam) or Texas Red-X goat anti-mouse #T6390 (Invitrogen), respectively, were incubated for 1 h. Stained cells were analyzed with the ZOE^*TM*^ Fluorescent Cell Imager (Bio-Rad) as described above (Figures 1B,C).

For analysis of mineralization, 2% alizarin solution was added for 20 min, followed by 5 washing cycles with ddH_2_O. Cells were analyzed by light microscopy (Axioskop 2, Axiocam MRc, Axiovision 4.7/ AutMess, Zeiss) (Figure 1D).

For experiments phAOBs were used between passages three and five, seeded on 6-well cell culture plates (Cellstar^Ⓡ^, Greiner Bio-One) and grown to 80% confluence.

hPDL fibroblasts were subjected to physiologic sustained mechanical load (2 g/cm^2^) or constant overload (8 g/cm^2^) for 16 or 24 h using glass weights with or without the autophagy inhibitor 3-Methyladenine (3-MA, 5 mM, Sigma-Aldrich). Pharmacological induction of autophagy by addition of rapamycin (50 nM, Enzo Life Sciences, Farmingdale, NY, USA) served as positive control. Rapamycin and 3-MA were added 1 h prior to mechanical stimulation.

phAOBs were cultured 24 h with supernatant from stimulated hPDL fibroblasts with or without additional soluble IL-6-receptor (sIL-6R, 100 ng/ml, Abcam, Cambridge, United Kingdom).

### 2.2. Autophagosome Quantification

Autophagosomes were quantified in hPDL fibroblasts using the Cyto-ID^Ⓡ^ Autophagy Detection Kit (#ENZ-51031, Enzo Life Sciences, Farmingdale, NY, USA) as described earlier (Memmert et al., 2019; Blawat et al., 2020). In short, cells were stimulated with load, overload or rapamycin and with or without 3-MA (Sigma-Aldrich). After 16 h of stimulation, cells were carefully detached from the bottom of the well with trypsin-EDTA (Biochrom GmbH, Berlin, Germany). Detached cells were stained according to the manufacturer's instructions with Cyto-ID^Ⓡ^ Green Detection Reagent (Enzo Life Sciences) for 30 min in the dark at room temperature. Stained autophagosomes were measured by flow cytometry (BD FACSCalibur, BD Biosciences, Franklin Lakes, NJ, USA) and analyzed using Flowing Software (http://flowingsoftware.btk.fi/).

### 2.3. Analysis of Protein Expression

In order to obtain information about the influence of autophagy on cytokine expression under mechanical stimulation, a simultaneous analysis of several proinflammatory cytokines in stimulated hPDL fibroblast culture supernatants at 16 h was performed by a commercially available enzyme-linked immunoassay (ELISA) kit. The Human Common Cytokines Multi-Analyte ELISArray^*TM*^ Kit (MEH-004A, Qiagen, Hilden, Germany) was used according to the manufacturer's instructions. Absorbance was measured with a microplate reader (PowerWave x, BioTek Instruments, Winooski, VT, USA) at 450 nm with a 570 nm correction wavelength. For validation of regulated IL-6 protein expression, protein-concentration of IL-6 in hPDL fibroblast culture supernatants after 16 and 24 h stimulation was analyzed by a commercially available enzyme-linked immunoassay (ELISA) kit (IL6 Single Analyte ELISA Kit, SEH00560A, Qiagen). The protocol provided by the manufacturer was performed as followes: absorbance was assessed with the same microplate reader (PowerWave x) at the same conditions as mentioned above (450 nm with a 570 nm correction wavelength). Cell number was counted, using an automatic cell counter (Moelab, Hilden, Germany), and used for data normalization.

### 2.4. Analysis of Gene Expression

Gene expressions of *il-6* and membrane-bound proteases *adam10* and *adam17* (both purchased from Qiagen) in hPDL fibroblasts as well as expressions of *opg* and *rankl* in phAOBs were analyzed by real-time polymerase chain reaction (rtPCR). RNA was isolated using the commercially available RNeasy Protect Mini Kit (Qiagen) according to the manufacturer's instructions. RNA concentration was measured using a NanoDrop^Ⓡ^ND-1000 spectrophotometer at 260 nm wavelength (NanoDrop Technologies, Wilmington, DE, USA) and consecutively transcribed to cDNA using the iScript^*TM*^ Select cDNA Synthesis Kit (Bio-Rad Laboratories, Munich, Germany). Afterwards, 1 μl of cDNA, 2.5 μl of the specific primer (Metabion, Martinsried, Germany or Qiagen according to Table 1), 12.5 μl of QuantiTect SYBR Green Master Mix (Qiagen) and 9 μl of nuclease free water were mixed and gene expression was detected in the iCycler iQ^*TM*^ real-time PCR detection system (Bio-Rad Laboratories). Protocol for rtPCR was as follows: an initial denaturation step at 95°C for 5 min was followed by 40 cycles of 10 s at 95°C, 30 s at annealing temperatures specific for the primers, and 30 s at 72°C for elongation. *Glycerinaldehyd-3-phosphate dehydrogenase* (*gapdh*) in phAOBs and *ribosomal protein L22* (*rpl22*) in hPDL fibroblasts served as housekeeping genes for data normalization.

**Table 1:** List of primers used for rtPCR.

**Gene**	**Primer sequences**	**Annealing temperature (^**°**^C)**
Adam10	(Primer was purchased from Qiagen)	60
Adam17	(Primer was purchased from Qiagen)	60
Gapdh	5′-CAC TCC TCC ACC TTT GAC GC-3′ 3′-CCA CCA CCC TGT TGC TGT A-5′	60
Il-6	5′-CAG GAG CCC AGC TAT GAA CT-3′ 3′-AGC AGG CAA CAC CAG GAG-5′	60
Opg	5′-TGC AGT ACG TCA AGC AGG AGT G-3′ 3′-TCC AGC TTG CAC CAC TCC AAA TC-5′	66
Rankl	5′-GCC AGT GGG AGA TGT TAG-3′ 3′-TTA GCT GCA AGT TTT CCC-5′	55
Rpl22	5′-TGA TTG CAC CCA CCC TGT AG-3′ 3′-GGT TCC CAG CTT TTC CGT TC-5′	60

### 2.5. Statistical Analysis

Experiments were repeated at least three times. Descriptive analyses of data were presented as means ± standard errors of the mean (SEM). Statistical analyses were performed with GraphPad Prism (Version 7.00 for Windows, GraphPad Software, San Diego, California USA, www.graphpad.com). Multiple comparisons were conducted using Kruskal-Wallis test followed by Dunnett's *T*-tests. Significance between two groups was calculated by the Mann-Whitney-Test. *P* < 0.05 were considered statistically significant.

## 3. Results

### 3.1. Effects of 3-MA on Mechanical Stimulation of Autophagy

**Figure 2:**
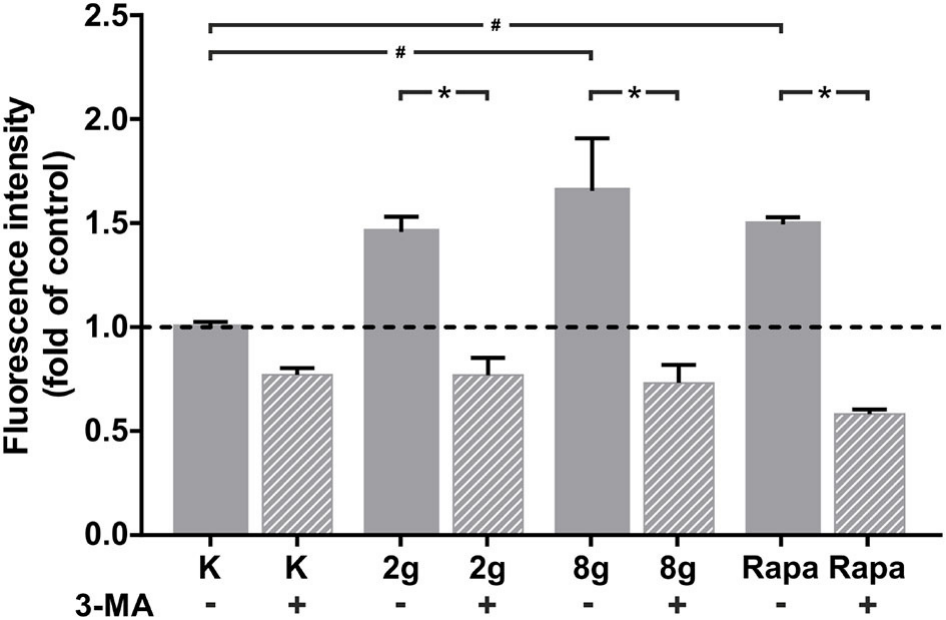
Autophagosome accumulation, indicated by fluorescence, after 16 h of treatment with 2 g/cm^2^, 8 g/cm^2^ or Rapamycin (Rapa; 50 nM) with or without autophagy inhibitor 3-MA. Untreated cells served as control. Unstained cells served as negative control (not shown). Fluorescence intensity is shown as fold of control. Mean ± SEM (*n* = 8 replicates); # significant (*p* < 0.05) difference between groups as determined by Dunnett's *T*-test. ^*^ significant (*p* < 0.05) difference between two groups (uninhibited and inhibited) as determined by the Mann-Whitney-Test.

Induction of autophagy was measured by the accumulation of stained autophagosomes in hPDL fibroblasts after stimulation with sustained mechanical stress. Pharmacological induction of autophagy via rapamycin served as positive control, whereas unstained hPDL fibroblasts served as negative control. Mechanical overload of 8 g/cm^2^, as well as the addition of rapamycin, for 16 h led to a significant increase in autophagosome accumulation, whereas stimulation with physiological pressure of 2 g/cm^2^ did not lead to significant changes in fluorescence intensity. Addition of 3-MA to stimulated cells reliably inhibited autophagosome formation and led to a significant decrease in fluorescence intensity in all groups assessed (Figure 2).

### 3.2. Effects of Autophagy on Mechanical Induced IL-6 Expression in hPDL Fibroblasts

**Figure 3:**
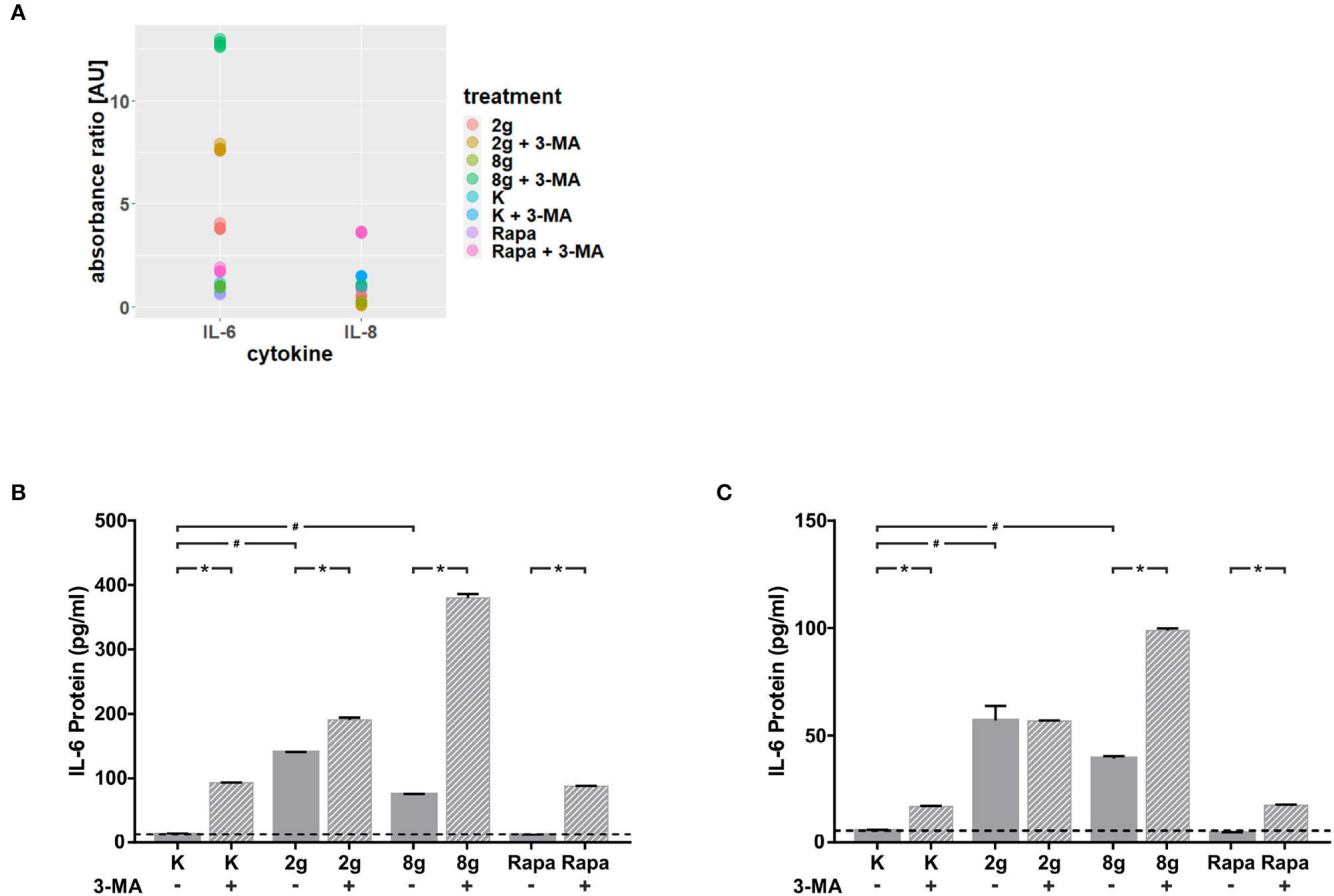
**(A)** Absorbance ratios of examined cytokines with control groups above detection limit. Effects of mechanical stimulation (2 or 8 g/cm^2^) or Rapamycin (Rapa; 50 nM) on cytokine levels in supernatants with or without autophagy inhibitor 3-MA at 16 h as determined by the Human Common Cytokines Multi-Analyte ELISArray^*TM*^ Kit (Qiagen). Untreated cells served as control. **(B)** Effects of mechanical stimulation (2 or 8 g/cm^2^) or Rapamycin (Rapa; 50 nM) on IL-6 protein level in supernatants with or without autophagy inhibitor 3-MA at 16 h as determined by ELISA. Untreated cells served as control. Mean ± SEM (*n* = 12); # significant (*p* < 0.05) difference between groups as determined by Dunnett's *T*-test. ^*^ significant (*p* < 0.05) difference between two groups (uninhibited and inhibited) as determined by the Mann-Whitney-Test. **(C)** Effects of mechanical stimulation (2 or 8 g/cm^2^) or Rapamycin (Rapa; 50 nM) on IL-6 protein level in supernatants with or without autophagy inhibitor 3-MA at 24 h as determined by ELISA. Untreated cells served as control. Mean ± SEM (*n* = 12); # significant (*p* < 0.05) difference between groups as determined by Dunnett's *T*-test. ^*^ significant (*p* < 0.05) difference between two groups (uninhibited and inhibited) as determined by the Mann-Whitney-Test.

**Figure 4:**
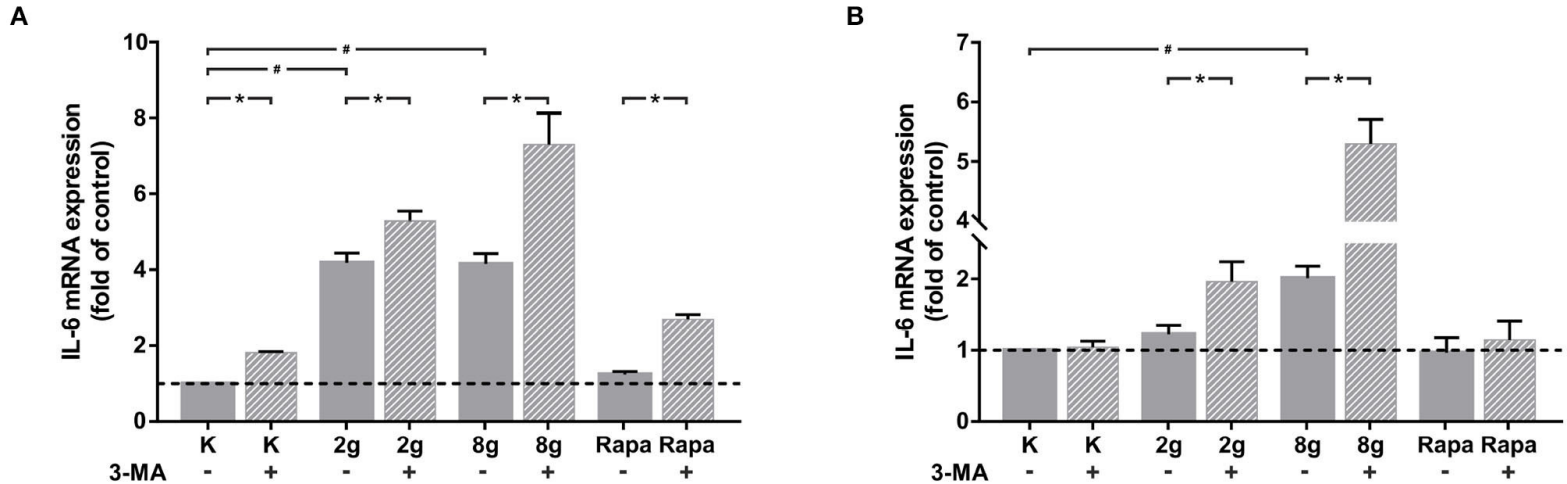
**(A)** Effects of mechanical stimulation (2 or 8 g/cm^2^) or Rapamycin (Rapa; 50 nM) with or without autophagy inhibitor 3-MA on *il-6* gene expression at 16 h as determined by rtPCR. Untreated cells served as control. Mean ± SEM (*n* = 6); # significant (*p* < 0.05) difference between groups as determined by Dunnett's *T*-test. ^*^ significant (*p* < 0.05) difference between two groups (uninhibited and inhibited) as determined by the Mann-Whitney-Test. **(B)** Effects of mechanical stimulation (2 or 8 g/cm^2^) or Rapamycin (Rapa; 50 nM) with or without autophagy inhibitor 3-MA on *il-6* gene expression at 24 h as determined by rtPCR. Untreated cells served as control. Mean ± SEM (*n* = 6); # significant (*p* < 0.05) difference between groups as determined by Dunnett's *T*-test. ^*^ significant (*p* < 0.05) difference between two groups (uninhibited and inhibited) as determined by the Mann-Whitney-Test.

We were able to obtain initial indications of how cytokine expression was affected by mechanically induced autophagy using the Human Common Cytokines Multi-Analyte ELISArray^*TM*^ Kit (Figure 3A and Table 2). On the basis of these preliminary experiments, focus was set on targeting IL-6, the protein regulated most strongly, with a commercially available ELISA-Assay. IL-6 was significantly upregulated in hPDL fibroblasts subjected to both magnitudes of mechanical stress for 16 (Figure 3B) and 24 h (Figure 3C). Induction of autophagy via rapamycin showed no effect on IL-6-levels. Autophagy inhibition, however, led to significant increases in IL-6 protein levels in nearly all tested groups. One exception was the stimulation of physiologic sustained mechanical load over 24 h, where 3-MA addition did not enhance IL-6 levels any further. IL-6 showed a similar pattern of expression in the supernatant at both time points, although the overall amount measured at 24 h appeared to be lower. Gene expression of *il-6* was also significantly upregulated by sustained mechanical stress after 16 h in hPDL fibroblasts (Figure 4A). Similar to results obtained on protein level, induction of autophagy via rapamycin had no significant effect on *il-6* gene expression. But again, autophagy inhibition resulted in a significant increase of *il-6* levels in all groups. After 24 h, a significant increase in *il-6* gene expression was detected in the overload group (Figure 4B). Again, chemical induction of autophagy had no effect on *il-6* gene expression, whereas inhibition of autophagy again elicited an increase in *il-6* gene expression in the mechanically simulated groups.

**Table 2:** Absorbance values of tested cytokines.

**Cytokine**	**Group**	**Absorbance (mean)**	**Cytokine**	**Group**	**Absorbance (mean)**
GM-CSF	K	NA	IL-1β	K	NA
GM-CSF	K+3-MA	NA	IL-1β	K+3-MA	NA
GM-CSF	Rapa	NA	IL-1β	Rapa	NA
GM-CSF	Rapa+3-MA	NA	IL-1β	Rapa+3-MA	NA
GM-CSF	LD	0,171	IL-1β	LD	0,087
GM-CSF	LD+3-MA	NA	IL-1β	LD+3-MA	NA
GM-CSF	8g	NA	IL-1β	8g	NA
GM-CSF	8g+3-MA	NA	IL-1β	8g+3-MA	NA
IFN-γ	K	NA	IL-2	K	NA
IFN-γ	K+3-MA	NA	IL-2	K+3-MA	NA
IFN-γ	Rapa	NA	IL-2	Rapa	NA
IFN-γ	Rapa+3-MA	NA	IL-2	Rapa+3-MA	NA
IFN-γ	LD	0,069	IL-2	LD	0,123
IFN-γ	LD+3-MA	NA	IL-2	LD+3-MA	NA
IFN-γ	8g	NA	IL-2	8g	NA
IFN-γ	8g+3-MA	NA	IL-2	8g+3-MA	NA
IL-10	K	NA	IL-4	K	NA
IL-10	K+3-MA	NA	IL-4	K+3-MA	NA
IL-10	Rapa	NA	IL-4	Rapa	NA
IL-10	Rapa+3-MA	NA	IL-4	Rapa+3-MA	NA
IL-10	LD	0,050	IL-4	LD	0,072
IL-10	LD+3-MA	NA	IL-4	LD+3-MA	0,079
IL-10	8g	NA	IL-4	8g	NA
IL-10	8g+3-MA	NA	IL-4	8g+3-MA	NA
IL-12	K	NA	**IL-6**	**K**	**0,041**
IL-12	K+3-MA	NA	**IL-6**	**K+3-MA**	**NA**
IL-12	Rapa	NA	**IL-6**	**Rapa**	**NA**
IL-12	Rapa+3-MA	0,113	**IL-6**	**Rapa+3-MA**	**0,072**
IL-12	LD	0,254	**IL-6**	**LD**	**0,157**
IL-12	LD+3-MA	NA	**IL-6**	**LD+3-MA**	**0,312**
IL-12	8g	NA	**IL-6**	**8g**	**0,040**
IL-12	8g+3-MA	NA	**IL-6**	**8g+3-MA**	**0,520**
IL-17A	K	NA	**IL-8**	**K**	**0,478**
IL-17A	K+3-MA	NA	**IL-8**	**K+3-MA**	**0,722**
IL-17A	Rapa	NA	**IL-8**	**Rapa**	**0,416**
IL-17A	Rapa+3-MA	NA	**IL-8**	**Rapa+3-MA**	**1,732**
IL-17A	LD	0,085	**IL-8**	**LD**	**0,251**
IL-17A	LD+3-MA	NA	**IL-8**	**LD+3-MA**	**0,040**
IL-17A	8g	NA	**IL-8**	**8g**	**0,129**
IL-17A	8g+3-MA	NA	**IL-8**	**8g+3-MA**	**0,500**
IL-1α	K	NA	TNF-α	K	NA
IL-1α	K+3-MA	NA	TNF-α	K+3-MA	NA
IL-1α	Rapa	NA	TNF-α	Rapa	NA
IL-1α	Rapa+3-MA	NA	TNF-α	Rapa+3-MA	NA
IL-1α	LD	NA	TNF-α	LD	NA
IL-1α	LD+3-MA	NA	TNF-α	LD+3-MA	NA
IL-1α	8g	NA	TNF-α	8g	NA
IL-1α	8g+3-MA	NA	TNF-α	8g+3-MA	NA

*Effects of mechanical stimulation (2 g/cm^2^ or 8 g/cm^2^) or Rapamycin (Rapa; 50 nM) on cytokine levels in supernatants with or without autophagy inhibitor 3-MA at 16 h as determined by the Human Common Cytokines Multi-Analyte ELISArray^TM^ Kit (Qiagen). Untreated cells served as control. If the values were below the detection limit specified by the manufacturer, the value is indicated in the table with NA. Cytokines whose control groups were above the detection limit are highlighted in bold font*.

### 3.3. Effects of Autophagy and Mechanical Stimulation on Cell-Cell Communication

**Figure 5:**
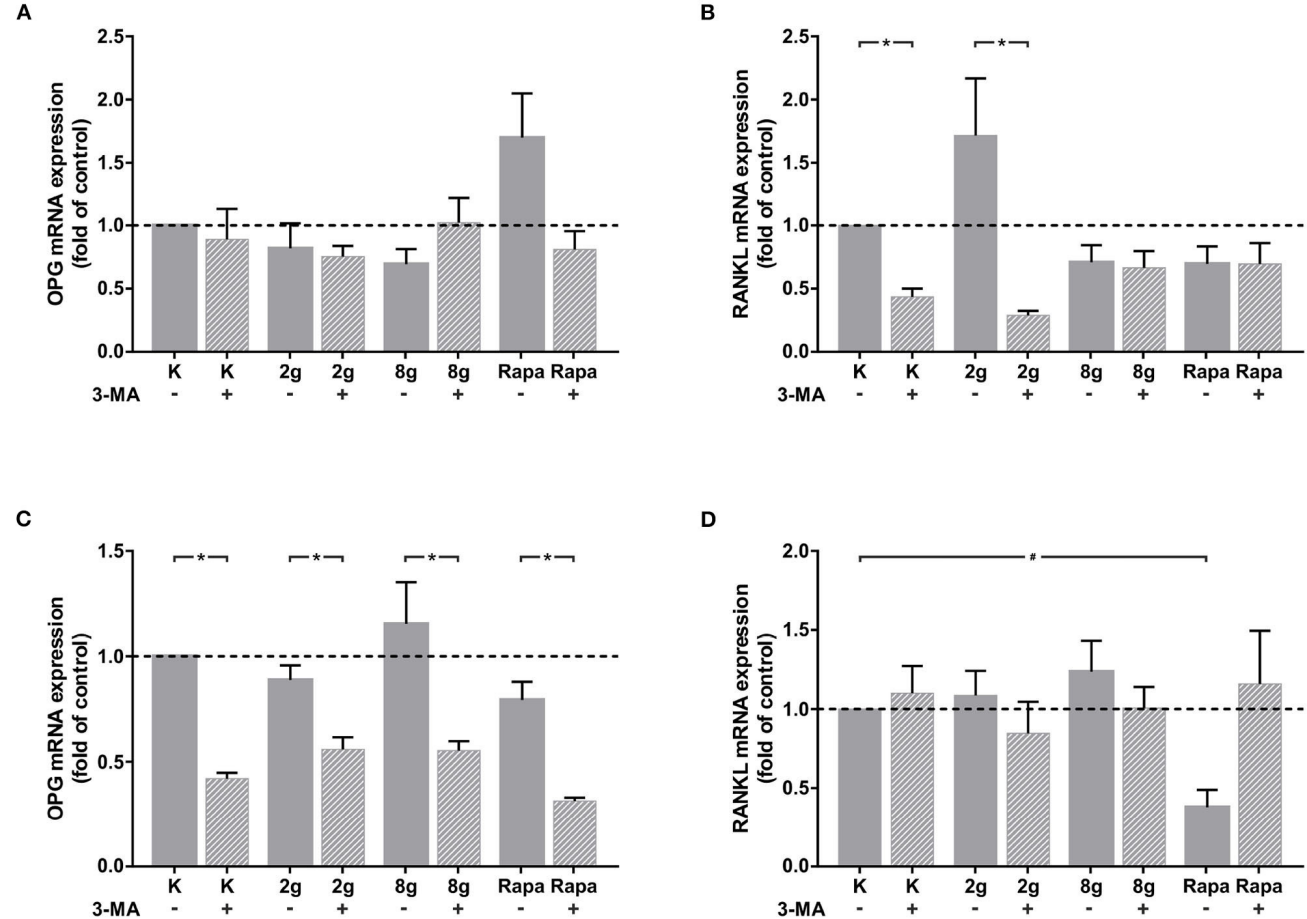
**(A)** Effects on *opg* gene expression in phAOBs after 24 h cultivation without sIL-6R and with supernatant from hPDL fibroblasts with or without autophagy inhibitor 3-MA, treated with mechanical stimulation (2 or 8 g/cm^2^) or Rapamycin (Rapa; 50 nM) for 16 h. Mean ± SEM (*n* = 6); # significant (*p* < 0.05) difference between groups as determined by Dunnett's *T*-test. ^*^ significant (*p* < 0.05) difference between two groups (uninhibited and inhibited) as determined by the Mann-Whitney-Test. **(B)** Effects on *rankl* gene expression in phAOBs after 24 h cultivation without sIL-6R and with supernatant from hPDL fibroblasts with or without autophagy inhibitor 3-MA, treated with mechanical stimulation (2 or 8 g/cm^2^) or Rapamycin (Rapa; 50 nM) for 16 h. Mean ± SEM (*n* = 6); # significant (*p* < 0.05) difference between groups as determined by Dunnett's *T*-test. ^*^ significant (*p* < 0.05) difference between two groups (uninhibited and inhibited) as determined by the Mann-Whitney-Test. **(C,D)** Panels show the graphs respective to **(A,B)** with addition of sIL-6R.

In order to analyze the effect that autophagy exerts on cell-cell communication via the regulation of IL-6 expression, phAOBs were cultured with supernatant of stimulated hPDL fibroblasts with and without the addition of sIL-6R. Gene expression of *rankl* and *opg* was analyzed in phAOBs cultured without additional sIL-6R (Figures 5A,B). Without the receptor, *opg* expression did not change significantly (Figure 5A). Interestingly, even in the absence of the receptor, autophagy inhibition in hPDL fibroblasts under control and physiological conditions significantly reduced *rankl* gene expression (Figure 5B). The addition of sIL-6R to hPDL fibroblast supernatant led to a marked effect on *opg* gene expression in phAOBs (Figure 5C). Inhibition of autophagy resulted in a significant decrease of *opg* gene expression in all groups assessed. However, application of mechanical pressure to hPDL fibroblasts as well as autophagy induction resulted in no significant changes in *opg* expression levels. *Opg* antagonist *rankl* showed almost no response to the addition of soluble IL-6 receptor (Figure 5D). A significant decrease in *rankl* expression in phOABs was observed when autophagy was pharmacologically induced in hPDL fibroblasts by rapamycin.

### 3.4. Effects of Autophagy and Mechanical Stimulation on the Expression of ADAM Proteases

**Figure 6:**
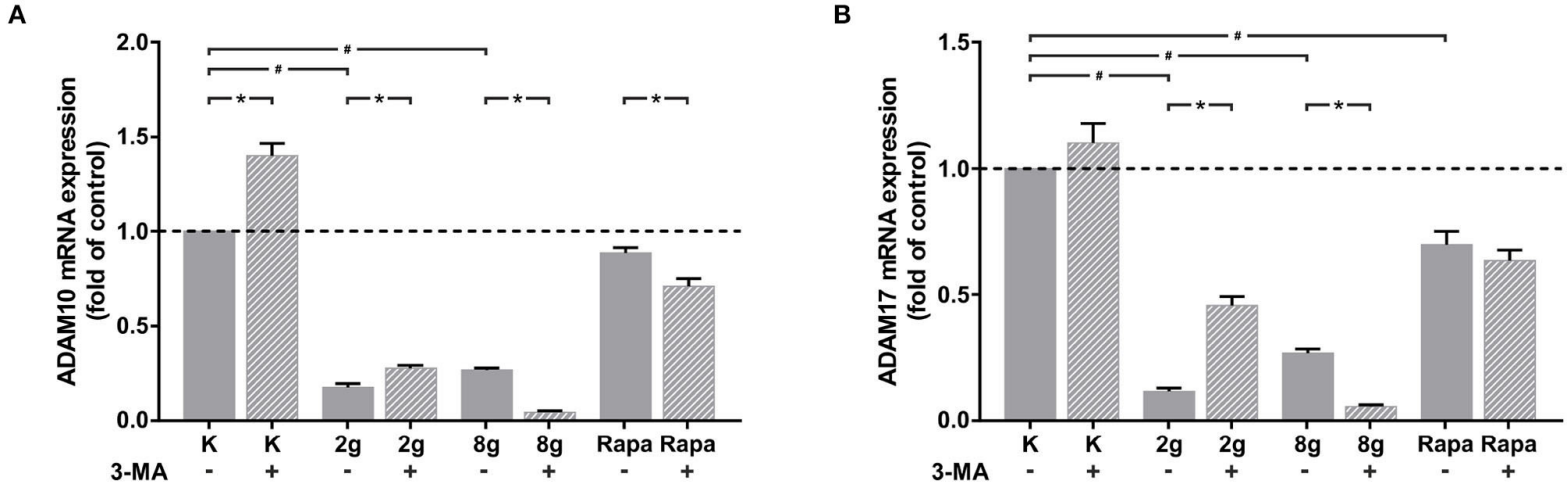
**(A)** Effects of mechanical stimulation (2 g/cm^2^ or 8 g/cm^2^) or Rapamycin (Rapa; 50 nM) with or without autophagy inhibitor 3-MA on *adam10* gene expression at 16 h as determined by rtPCR. Untreated cells served as control. Mean ± SEM (*n* = 6); # significant (*p* < 0.05) difference between groups as determined by Dunnett's *T*-test. ^*^ significant (*p* < 0.05) difference between two groups (uninhibited and inhibited) as determined by the Mann-Whitney-Test. **(B)** Effects of mechanical stimulation (2 or 8 g/cm^2^) or Rapamycin (Rapa; 50 nM) with or without autophagy inhibitor 3-MA on *adam17* gene expression at 16 h as determined by rtPCR. Untreated cells served as control. Mean ± SEM (*n* = 6); # significant (*p* < 0.05) difference between groups as determined by Dunnett's *T*-test. ^*^ significant (*p* < 0.05) difference between two groups (uninhibited and inhibited) as determined by the Mann-Whitney-Test.

The effects of mechanical stimulation as well as autophagy on the expression of ADAM proteases, responsible for cleavage of the IL-6 receptor from cell surfaces, were also investigated in hPDL fibroblasts. The expression of both ADAM protases, *adam10* (Figure 6A) and *adam17* (Figure 6B), were significantly inhibited by both magnitudes of mechanical pressure as well as by autophagy induction. Interestingly, autophagy inhibition under control and physiological conditions resulted in a significant increase in gene expression of both ADAM proteases. Autophagy inhibition under overload, on the other hand, led to a significant decrease in gene expression of both ADAM proteases. Autophagy inhibition following chemical autophagy induction also reduced *adam10* expression levels significantly (Figure 6A), whereas *adam17* levels showed no significant changes (Figure 6B).

## 4. Discussion

The present study adressed the influence of autophagy on IL-6 production in hPDL fibroblasts during mechanical load and overload and addresses the consequences of this regulation with respect to osteoclastogenesis *in vitro*.

The autophagic response was dose-dependent in respect to mechanical pressure stimulation and was shown to be reliably inhibited by 3-MA. Our results suggest that mechanical pressure stimulation leads to a significant upregulation of IL-6 expression on gene and protein level. Autophagy inhibition further enhanced the effects of mechanical stimulation on IL-6 expression. These regulations also had consequences on cell-cell communication. Inhibition of autophagy in hPDL fibroblasts restrained *opg* gene expression in phAOBs via IL-6 secretion. Although being interrelated, we could not demonstrate an IL-6 related effect on the expression of *rankl*. We further found that the expression of proteases *adam10* and *adam17* were significantly downregulated in hPDL fibroblasts in response to mechanical stimulation. The response to autophagy inhibition seemed to be dependent on stimulus intensity. Inhibition of autophagy in combination without additional stress or physiological load led to upregulation in *adam* expressions. Enhanced stress stimulation due to overload and chemical induction of autophagy in combination with autophagy inhibition led to reduced *adam* expressions.

We demonstrated in the current as well as in earlier studies that autophagy in hPDL fibroblasts is regulated by pressure in a dose-dependent manner (Blawat et al., 2020). Autophagy is an important adaptive mechanism to mechanical forces in periodontal cells and tissues, this has already been shown by our group and others (Memmert et al., 2019, 2020; Blawat et al., 2020; Chen and Hua, 2021). In a previous study by our group, we focused on how autophagy is activated by stimulation with mechanical load and overload. For qualitative protein phosphorylation profiling of the mTOR signaling pathway, a human mTOR Signaling Phospho Specific Antibody Array was used. The influence of mechanical stimulation on the PI3K/AKT pathway was shown to be dose dependent. We were able to identify phosphorylation sites Thr308 and Thr474 of AKT pathway to be influenced by mechanical loading. Our results suggest at least a partial influence of mTOR on autophagy regulation via the PI3K/AKT signal pathway (Blawat et al., 2020). In this study, we focused on the effects that autophagy excites under mechanical stimulation in hPDL fibroblasts. Therefore, it was important to show in advance that with our pharmacological inhibitors and inducers, autophagy is reliably affected in hPDL fibroblasts.

The consequences of autophagy regulation and dysregulation under mechanical stimulation, especially with respect to cell-cell communication, are still unclear. Cytokines are important cell messengers and play a decisive role in cellular interactions. IL-6 is a central mediator of the body's systemic defense response to injury. It is produced in vast amounts by various cell types and reaches high circulatory levels. Its main roles include regulation of immune and inflammatory responses, hematopoiesis, control of acute phase protein synthesis and influence on bone metabolism (Garman et al., 1987; Lotz et al., 1988; Gauldie et al., 1992; Ogawa, 1992). Bone metabolism relies on the interplay of bone formation by osteoblasts and resorption by osteoclasts. IL-6 appears to stimulate resorption of bone and also regulate the activity of both cell types (Tamura et al., 1993; Xiong et al., 2011, 2015). IL-6 has been suggested to be involved in the pathogenesis of osteoporosis after estrogen loss via stimulation of osteoclast differentiation. Estrogens have been shown to inhibit IL-6 production (Jilka et al., 1992; Poli et al., 1994). We demonstrated that the production of IL-6 by hPDL fibroblasts is affected by mechanical pressure *in vitro* at both the gene and protein levels. Our research group has previously shown that inducing OTM using a 25 gram nickel-titanium coil, over a period of 3 days, resulted in an increased expression of IL-6 in rats. However, this upregulation was not found to be statistically significant (Rath-Deschner et al., 2021). A significant increase in IL-6 expression was shown in OTM experiments with mice using higher force intensities (Mo and Hua, 2018). Using our *in vivo* experimental setup to simulate physiological pressure and overload on hPDL fibroblasts during OTM, we were able to demonstrate that after both 16 and 24 h of cell stimulation, a significant increase in IL-6 expression occurred at both magnitudes of mechanical stimulation. Tension on the other hand had no significant effect on IL-6 levels, as demonstrated earlier (Rath-Deschner et al., 2021). Thus, our results suggest that the mode of mechanical stimulation rather than stimulation intensity influences the expression of this cytokine in hPDL fibroblasts.

In our *in vitro* setup, we were able to demonstrate that inhibition of autophagy using 3-MA during application of mechanical pressure causes a sharp increase of IL-6 expression in hPDL fibroblasts on gene and protein level. This observation is consistent with mouse experiments, where gene expression of inflammatory factors *il-1, il-6*, and *TNF-*α increased in periodontal tissues after concomitant administration of OTM and 3-MA (Mo and Hua, 2018; Chen and Hua, 2021). This response to inhibition suggests that autophagic processes may play a major role in controlling cell-cell communication, which was the focus of our study. Our findings expand and deepen the conclusions of the animal experiment. We were able to demonstrate that hPDL fibroblasts are, at least in part, responsible for the increase of IL-6 in periodontal tissues. One explanation for this observation could be that inhibition of autophagy deprives the cell of a key adaptive mechanism.

Since IL-6 plays an important role in cell-cell communication, we wanted to determine how sustained mechanical stress on hPDL fibroblasts affects osteoblast-osteoclast communication. IL-6 is thought to play a positive regulatory role in osteoclast differentiation (Palmqvist et al., 2002). However, for IL-6 interaction with its target cell, the so-called trans-signaling pathway is particularly important in addition to the classical signaling pathway. The latter is executed via a membrane-bound IL-6R on a few specific cells, like macrophages, neutrophils, hepatocytes and some type of T-cells. This trans-signaling pathway depends on cleaved sIL-6R, which enables signal transduction between IL-6 and the ubiquitous membrane protein gp130 (Rose-John and Heinrich, 1994; Rose-John et al., 2007; Scheller et al., 2011). In our experiments, supernatant of stimulated hPDL fibroblasts was initially added to phAOBs without sIL-6R. Subsequently, gene expression of *rankl* and *opg* were evaluated. As IL-6 signaling with osteoclasts requires interaction of IL-6 with its receptor, as expected, we did not see any effect on gene expression of *opg* in phAOBs. Therefore, all observed changes after addition of the receptor can be attributed to IL-6 secreted by hPDL fibroblasts. External addition of soluble IL-6 receptor resulted in significant decreases in *opg* expression by phAOBs in all of the groups studied. OPG functions as a decoy receptor for RANKL. By binding RANKL, OPG inhibits the activation of NF-κB, a central and fast-acting transcription factor for immune activation and differentiation of precursor cells into osteoclasts (Theoleyre et al., 2004). Our work sheds light on the background and underlying mechanisms at cellular level and highlights the cross-talk between fibroblasts and osteoblasts. Therefore, our results can be used as an explanatory model for findings of *in vivo* studies performed on mice, where micro-CT scanning demonstrated that inhibition of autophagy with simultaneous performance of OTM, resulted in a significant reduction in bone density (Chen and Hua, 2021).

When *rankl* expression was examined in stimulated phAOBs, it was found that autophagy inhibition in combination with no stress stimulus or physiological load to hPDL fibroblasts resulted in significant downregulation of *rankl* in phAOBs. However, because of the absence of soluble IL-6 receptor this effect does not appear to be dependent on IL-6-signaling. Further experimental studies are needed to investigate this observation in more detail. The only effect on *rankl* expression in phAOBs when sIL-6R was added was observed after pharmacologically induced autophagy in hPDL fibroblasts. As IL-6 was not upregulated by rapamycin stimulation in hPDL fibroblasts and a similar trend was observed without sIL-6R, we do not attribute this effect to IL-6 but to other processes triggered by autophagy induction, which will be investigated in further experiments. Interestingly in other *in vitro* studies IL-6/sIL-6R complex induced the expression of RANKL by mouse osteoblasts (Udagawa et al., 1995; Palmqvist et al., 2002). In these studies, osteoclast activity was controlled by osteoblasts through RANKL expression rather than OPG expression. However, our results suggest that in hPDL fibroblasts, osteoclast activity is more likely to be controlled via the reduction of OPG expression upon autophagy inhibition.

Next we investigated whether the expression of ADAM10 and ADAM17 is modulated by both pressure and autophagy. ADAMs play a crucial role in the trans-signaling pathway. They are able to cleave the membrane-bound IL-6 receptor, making its soluble form available, which enables signal transduction between IL-6 and the ubiquitous membrane protein gp130 (Müllberg et al., 1994; Matthews et al., 2003; Chalaris et al., 2007, 2010). ADAM10 is responsible for a slow and steady release of sIL-6R, whereas ADAM17 is responsible for rapid, short-term shedding of sIL-6R (Matthews et al., 2003). Interestingly, especially *adam10* was shown to be regulated by mechanical stimulation in a human chondrocytic cell line (Kobayakawa et al., 2016). Mechanical regulation of *adam17* has already been described in mechanically stretched rat cardiomyocytes (Niu et al., 2015). Since ADAMs influence the amount of soluble IL-6 receptor available, and ADAM17 has been shown to be strongly expressed in other periodontal cells – namely in the epithelium of gingival tissues – we investigated whether autophagy has an effect on mRNA expression of *adam10* or *adam17* in stimulated hPDL fibroblasts (Hirayama et al., 2017). We noted a significant decrease in mRNA expression for both *adam10* and *adam17* upon induction of autophagy by load, overload, and rapamycin. This was particularly evident in the pressure groups. Our results suggest that compression has a downregulatory effect on ADAM proteases while the existing literature contains evidence of upregulation of ADAM proteases during tensile strain (Niu et al., 2015; Kobayakawa et al., 2016). However, that ADAM17 plays a central role in the transduction of compressive stress has already been shown in murine tracheal epithelial cells (Shiomi et al., 2011). Autophagy inhibition in the control group and under physiological mechanical stress resulted in a significant increase in mRNA expression of both proteases. A significant inhibition, on the other hand, was observed under mechanical overload as well as induction of autophagy via rapamycin. This suggests that here again autophagy, depending on the size of the stress stimulus, has different regulatory effects on expression at different stress intensities through its bifurcated role as a cell protector and cell death inducer. To our knowledge, regulation of mRNA expression of *adam10* and *adam17* by mechanical load, overload and especially by autophagy have not been described in hPDL fibroblasts, yet. In the context of our study, our results suggest that the sIL-6R required for IL-6-induced osteoclast activation during OTM probably does not originate from hPDL fibroblasts. This suggests the involvement of other cell types in cell-cell communication of hPDL fibroblasts, phAOBs and osteoclasts. This interesting question will be investigated in further studies.

PDL fibroblasts, due to their position in the periodontium, are of particular importance for OTM and thus for the transmission of mechanical stimulation (de Araujo et al., 2014; Li et al., 2018; Memmert et al., 2019, 2020). Their central role makes it necessary to take a close look at them at cellular level in order to uncover their functions and the influence of important processes on their role in OTM. Therefore, we used established models for *in vitro* application of mechanical stress (Ullrich et al., 2019; Blawat et al., 2020). The approach of breaking down OTM to individual *in vitro* models offers the possibility of a differentiated view on different stress stimulations. This is particular relevant for autophagy analysis, as autophagy can be induced by different types of cellular stress, which are also involved in OTM (King, 2012; Li et al., 2018; Memmert et al., 2019, 2020). Especially hypoxia is a known autophagy inducer. To eliminate the risk of limited oxygen supply to hPDL fibroblasts in our study, pressure experiments were performed exclusively on dedicated cell culture wells with gas-permeable bottoms. This ensured proper oxygen supply to the cells at all times (Ullrich et al., 2019). Autophagy usually happens rapidly. We based the selection of our time points on an earlier study by our group, which focusses on autophagy regulation by mechanical load and overload. In this study we also included earlier time points, i.e. 4 h, but our results show that autophagy regulation was most pronounced after 16 h under mechanical stimulation in PDL fibroblasts (Blawat et al., 2020). Mechanical stimulation was performed by means of physiological load on the one hand as well as by overload on the other hand. In previous experiments we have used various load magnitudes for the mechanical stimulation of hPDL fibroblasts (Blawat et al., 2020). It has been reported that cells treated with a pressure of 4 g/cm^2^ suffer cell damage due to the applied excessive pressure (Kanzaki et al., 2002). In our experiments, however, increased cell death could only be reliably induced with the application of 8 g/cm^2^ (Blawat et al., 2020). Therefore, our previous results suggest, that stimulation with 8 g/cm^2^ exceeds a critical threshold and is rightfully termed overload. However, a load of 2 g/cm^2^ was established as physiological pressure in different studies and was also used in our experimental setup (Kanzaki et al., 2002; Schröder et al., 2018; Kirschneck et al., 2019; Blawat et al., 2020). In earlier experiments by our group a mechanical load of 2 g/cm^2^ appeared to have cell-protective effects (Blawat et al., 2020).

Pharmacologically, autophagy can be affected in terms of induction with the mTOR inhibitor rapamycin and inhibited by inhibitors of autophagosome formation such as 3-MA (Klionsky et al., 2016). To assess the influence of autophagy on cytokine expression and therefore on cell-cell communication a Cytokine Multi-Analyte ELISArray Kit, i.e. an unbiased approach was used. Since IL-6 was detected as the most strongly regulated cytokine, we further focused on the influence of autophagy on mechanically induced IL-6 expression. The upregulation of IL-6 protein expression due to autophagy inhibition under mechanical stimulation could be confirmed by ELISA and also on gene expression level by rtPCR. Consideration of other cytokines regulated by autophagy is beyond the scope of the manuscript and will therefore be addressed in separate studies. It should be further noted that we only analyzed the gene expression of ADAM proteases in our experiments. This is not to be equated with enzyme activity. However, other studies have also investigated ADAM10 and ADAM17 only on gene expression level, as evidence suggests that their gene expression correlates with their activity (Kinoshita et al., 2013; Cheng et al., 2021; Couselo-Seijas et al., 2021).

## 5. Conclusion

In summary, our results provide novel insights into the effects induced by autophagy regulation and deregulation in hPDL fibroblasts during mechanical load and overload. In response to pressure, IL-6 expression was induced in hPDL fibroblasts, which was further enhanced by autophagy inhibition. This led to a reduction in *opg* expression in phAOBs, which in turn could be responsible for the observed shift in the balance of bone formation and resorption during OTM under autophagy inhibition. While this could serve as an explanatory model for the accelerated tooth movement observed under autophagy inhibition, it could also represent a risk factor for unregulated bone loss.

## Data Availability Statement

The raw data supporting the conclusions of this article will be made available by the authors, without undue reservation.

## Ethics Statement

The studies involving human participants were reviewed and approved by Ethics Committee of the University of Bonn (#117/15 Memmert); primary human osteoblasts were explanted from alveolar bone fragments, which would have been discarded otherwise. Written informed consent to participate in this study was provided by the participants' legal guardian/next of kin.

## Author Contributions

AM, MW, JD, AJ, and SB-M: conceptualization. AM, JM, RBC, KB, BE, and SB-M: methodology. AM, BE, and SB-M: validation and formal analysis. AM, JW, and SB-M: investigation. AJ and SB-M: resources. AM, JM, BE, RBC, and SB-M: data curation. AM and SB-M: writing—original draft preparation. JW, JD, MW, and AJ: writing—review and editing. JD, MW, AJ, and SB-M: supervision. AJ, JD, and SB-M: project administration. All authors contributed to the article and approved the submitted version.

## Conflict of Interest

The authors declare that the research was conducted in the absence of any commercial or financial relationships that could be construed as a potential conflict of interest.

## Publisher's Note

All claims expressed in this article are solely those of the authors and do not necessarily represent those of their affiliated organizations, or those of the publisher, the editors and the reviewers. Any product that may be evaluated in this article, or claim that may be made by its manufacturer, is not guaranteed or endorsed by the publisher.
